# Non-small-cell lung cancer resectability: diagnostic value of PET/MR

**DOI:** 10.1007/s00259-014-2873-9

**Published:** 2014-08-15

**Authors:** Francesco Fraioli, Nicholas J. Screaton, Samuel M. Janes, Thida Win, Leon Menezes, Irfan Kayani, Rizwan Syed, Fulvio Zaccagna, Celia O’Meara, Anna Barnes, Jamshed B. Bomanji, Shonit Punwani, Ashley M. Groves

**Affiliations:** 1 Department of Nuclear Medicine and Radiology, University College London Hospitals NHS Foundation Trust, 235 Euston Road, London, NW1 2BU UK; 2 Department of Radiology, Papworth Hospital NHS Foundation Trust, Cambridge, CB23 3RE UK; 3 Lungs for Living Research Centre, UCL Respiratory Division of Medicine, University College London, London, WC1E 6JF UK; 4 Respiratory Medicine, Lister Hospital, Stevenage, UK; 5 Department of Radiological sciences, University of Rome “Sapienza”, Viale R. Elena 324, 00321 Rome, Italy

**Keywords:** Non-small-cell lung cancer, Lung cancer, PET/MR, TNM staging

## Abstract

**Purpose:**

To assess the diagnostic performance of PET/MR in patients with non-small-cell lung cancer.

**Methods:**

Fifty consecutive consenting patients who underwent routine ^18^F-FDG PET/CT for potentially radically treatable lung cancer following a staging CT scan were recruited for PET/MR imaging on the same day. Two experienced readers, unaware of the results with the other modalities, interpreted the PET/MR images independently. Discordances were resolved in consensus. PET/MR TNM staging was compared to surgical staging from thoracotomy as the reference standard in 33 patients. In the remaining 17 nonsurgical patients, TNM was determined based on histology from biopsy, imaging results (CT and PET/CT) and follow-up. ROC curve analysis was used to assess accuracy, sensitivity and specificity of the PET/MR in assessing the surgical resectability of primary tumour. The kappa statistic was used to assess interobserver agreement in the PET/MR TNM staging. Two different readers, without knowledge of the PET/MR findings, subsequently separately reviewed the PET/CT images for TNM staging. The generalized kappa statistic was used to determine intermodality agreement between PET/CT and PET/MR for TNM staging.

**Results:**

ROC curve analysis showed that PET/MR had a specificity of 92.3 % and a sensitivity of 97.3 % in the determination of resectability with an AUC of 0.95. Interobserver agreement in PET/MR reading ranged from substantial to perfect between the two readers (Cohen’s kappa 0.646 – 1) for T stage, N stage and M stage. Intermodality agreement between PET/CT and PET/MR ranged from substantial to almost perfect for T stage, N stage and M stage (Cohen’s kappa 0.627 – 0.823).

**Conclusion:**

In lung cancer patients PET/MR appears to be a robust technique for preoperative staging.

## Introduction

Non-small-cell lung cancer (NSCLC) accounts for 75 – 80 % of all lung cancers and is currently the leading cause of tumour-related deaths [1]. Accurate staging is critical in determining management. Surgical resection is the therapeutic standard in fit patients with stage I–IIIa disease, but unfortunately the majority of patients have advanced disease at presentation [2, 3]. Imaging plays a central role in establishing an accurate preoperative stage and directing further confirmatory tests. Thoracic contrast-enhanced (CE) CT and ^18^F-FDG PET/CT are performed to assess, respectively, morphology and metabolic TNM stage [4, 5]. CE CT has been widely used for the preoperative evaluation of tumour size and local extent, but is limited in differentiating malignant from reactive lymphadenopathy and in detecting distant metastatic disease [6]. FDG PET/CT has been reported to increase diagnostic accuracy in the differentiation of benign and malignant primary cancers and to improve identification of nodal disease and metastasis [7, 8].

MRI has been investigated as an emerging technique for whole-body cancer staging, providing high soft tissue contrast and potential for multiparametric tissue characterization through diffusion and dynamic CE sequences [9–11]. Previous studies assessed the feasibility of a whole-body MR protocol for lung cancer staging and demonstrated accuracy similar to that of PET/CT for NSCLC staging with better sensitivity than PET/CT for metastatic disease [12]. Combined PET/MR is a new technique that has shown similar performance to PET/CT in several, but generally small, retrospective studies [13, 16]. The potential advantages of the technique include multiparametric metabolic, functional and morphological information provided by combining radioactive tracers with different MR sequences. Furthermore, the lack of radiation using MRI is a major advantage over CT, particularly when repeat studies are used in patient management.

In this prospective study we compared the performance of PET/MR with conventional lung cancer staging in patients with potentially resectable disease.

## Materials and methods

### Patients

Our institutional ethics review board approved this prospective study, and informed consent was obtained from all patients.

From May 2012 to August 2013, 50 consecutive patients (28 men and 22 women; mean age 58 years, range 42 – 79 years) who underwent routine ^18^F-FDG PET/CT for potentially radically treatable lung cancer following a staging CT scan were recruited for PET/MR imaging on the same day. The patients were referred for PET scanning for enlarged nodes (25 patients), to help biopsy planning (8 patients), and for suspected M1a or M1b lesions (17 patients). The interval between CE CT and PET/CT was less than 14 days.

The PET/CT scan was performed in fasted patients using a Discovery STe 16-slice PETCT scanner (GE Healthcare, Milwaukee, WI) 60 min after intravenous injection of 5.5 MBq/kg of ^18^F-FDG. CT was performed according to a standard protocol with the following parameters: 140 keV, 30 – 170 mAs, pitch 1.75, and section thickness 3.75 mm (to match the PET section thickness). The mean (±standard deviation) PET/CT scan duration was 33 ± 4 min. The mean effective radiation dose from the PET/CT scan was 17.2 ± 2.7 mSv (9.2 ± 2.3 mSv from the PET scan).

Patients were transferred immediately to the PET/MR scanner (3 T Biograph mMR; Siemens, Erlangen, Germany) where a simultaneous PET and MR acquisition was performed approximately 20 min (15 – 35 min) after the PET/CT examination with 5 min per bed position). Four tissue class (soft tissue, fat, lung, air) attenuation correction maps were calculated from two-point Dixon sequences. The MR protocol for each patient included axial T2-weighted HASTE, axial diffusion (DWI) and axial T1-weighted 3-D interpolated breath-hold examination (VIBE) with fat suppression after contrast enhancement of the thorax and abdomen by administration of 0.2 ml/kg Gd.

### Reference standard

**Fig. 1:**
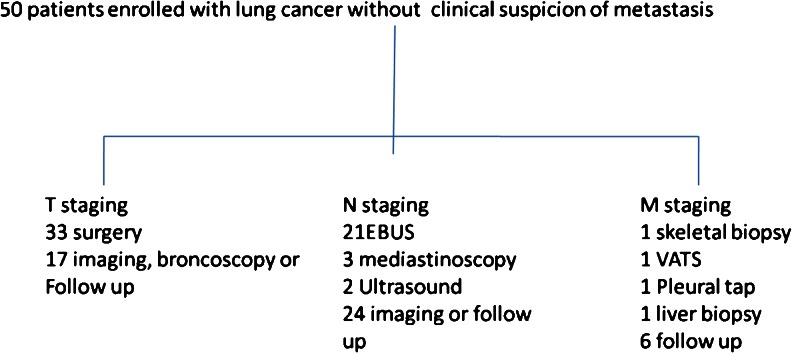
Flowchart showing how TNM stage was determined. *EBUS* endobronchial ultrasound, *Ultrasound* biopsy of supraclavicular nodes, *VATS* video-assisted thoracoscopic surgery

The reference standard was histology from thoracotomy in patients considered operable. Patients considered inoperable were staged using results from imaging, bronchoscopy, nodal assessment or follow up. Patients had standard care follow-up which included CE CT + PET/CT or MR when required. A flowchart of the reference standard TNM staging is shown in Fig. 1.

When preoperative CT and FDG PET/CT depicted enlarged or suspicious nodes, endobronchial ultrasonography (EBUS), mediastinoscopy or ultrasound-guided aspiration was performed. Patients with stage I, II or IIIA NSCLC were considered operable. Of the 50 patients, 33 were surgically treated, 14 underwent chemotherapy and 3 underwent radiotherapy. None underwent radical radiotherapy.

Tumour histology was consistent with large-cell carcinoma in 9 patients (18 %), adenocarcinoma in 28 patients (56 %) and squamous cell carcinoma in the remaining 13 patients (26 %). Histology was obtained preoperatively in 43 patients from percutaneous needle aspiration biopsy (14 patients), EBUS (18 patients), bronchoscopy (8 patients), pleural effusion (1 patient), lymph node mediastinoscopy (1 patient), and supraclavicular lymph node ultrasound-guided aspiration (1 patient). Seven patients with highly suspicious small lesions that were classified as stage I by the reference standard underwent surgery prior to preoperative histological assessment. Surgical pathological T stage was available in 33 patients, from thoracotomy (lobectomy in 25 patients, bilobectomy in 4, and results in 1) in 30 patients and from video-assisted thoracoscopic surgery resection in 3 patients.

In 17 inoperable patients (14 with stage IV disease and 3 inoperable for medical reasons) T stages were determined in consensus using imaging, bronchoscopy or follow-up results. N stages were determined preoperatively with EBUS in 21 patients, lymph node mediastinoscopy in 3 patients, and supraclavicular lymph node aspiration in 2 patients. In the 33 patients who underwent thoracotomy, nodal dissection was performed. In the remaining patients, nodes were assessed by imaging and follow-up. Metastatic lesions were confirmed on pathological examination in four patients. Six patients had metastatic lesions confirmed on specific organ-dedicated or follow-up imaging studies.

### Image analysis

PET/MR data were anonymized and sent to a workstation(OsiriX, http://www.osirix-viewer.com/) for evaluation. Images were interpreted by a chest radiologist with nuclear medicine training (F.F, 10 years) and by a radiologist with dedicated skills in MRI and also with experience in interpreting nuclear medicine studies (S.P, 10 years). Images were interpreted first separately and then discordant findings were resolved by consensus. Readers were unaware of the clinical and pathological results and imaging findings of the other modalities. PET/MR image quality was scored subjectively on a scale of 1 to 3 (*1* unacceptable, *2* good, *3* excellent).

Lung cancer staging was performed following the TNM classification (7th edition). Readers were free to evaluate either MR images alone or fused PET/MR images in a real time approach. Each reader recorded:

1. T stage, lesion dimension and nodal status.
2. A confidence score for each site of nodal and distant disease (*1, 2* disease unlikely, *3* possible disease, *4* probable disease, *5* likely disease, and *6* definite disease). Lymph node stations were allocated to 22 groups according to lymph node map definitions for lung cancer staging recommended by the International Association for the Study of Lung Cancer.
3. Distant metastases, divided into 28 sites, recording the category (M1a or M1b) and the anatomical location.

PET/CT images were separately reviewed for TNM staging without knowledge of the PET/MRI findings by two different readers (L.M., R.Z.), each with more then 5 years experience in PET.

### Statistical analysis

PET/MR imaging findings were recorded in an electronic database (Microsoft Excel 2008 for Macintosh; Microsoft, Redmond, WA) and statistical analysis was performed using dedicated software (SPSS 13.0 for Macintosh; SPSS, Chicago, IL).

ROC curve analysis was performed to assess the accuracy, sensitivity and specificity of PET/MR in assessing TNM stage. For statistical analysis when dichotomization of data was required, we considered 1 – 3 as negative for the presence of disease and 4 – 6 as indicative of the presence of disease. A *p* value <0.05 was considered statistically significant.

The generalized kappa statistic (κ) was used to determine PET/MR interobserver agreement and intermodality agreement (PET/CT vs. PET/MR) for TNM staging considering: κ <0.2 as slight agreement, κ 0.21 – 0.40 as fair agreement, κ 0.41 – 0.60 as moderate agreement, κ 0.61 – 0.80 as substantial agreement, and κ 0.81 – 1.00 as almost perfect agreement [17].

## Results

**Fig. 2:**
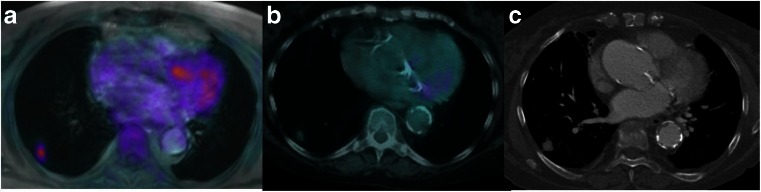
Axial PET/MR image (**a**) PET/CT image (**b**) and CE CT image (**c**) in a 63-year-old woman. A small nodule shows FDG uptake on both the PET/MR and PET/CT images

PET/MR examinations yielded diagnostic image quality from good to excellent in all patients (Fig. 2). The mean PET/MR scan time (including room time) was 38 ± 9 min.

According to the reference standard, 17 of the 50 patients (34 %) were classified as stage I, 12 (24 %) were classified as stage II, 8 (16 %) were classified as stage IIIA and 13 (26 %) were classified as stage IIIb and IV (Table 1).Agreement in T, N and M staging between reader 1 and reader 2 was substantial to excellent (Table 2). The staging results showed a high concordance between PET/MR and the reference in most patients. The details of the staging assessments are shown in Tables 3, 4 and 5.

**Table 1:** Staging according to the reference and PET/MR. The values presented are number (%)

Stage	Reference	PET/MR
I	17/50 (34)	14/50 (28)
II	12/50 (24)	17/50 (34)
IIIa	8/50 (16)	6/50 (12)
IIIb-IV	13/50 (26)	13/50 (26)

**Table 2:** Agreement in T, N and M staging between reader 1 and reader 2 (Cohen's κ)

	Cohen’s κ	Standard deviation	*p* value
T	0.753	0.069	0.000
N	0.646	0.089	0.000
M	1.000	0.000	0.000

**Table 3:** T staging according to the reference and PET/MR

PET/MR	Reference			
	T1	T2	T3	T4
T1	12	0	2	1
T2	1	11	2	4
T3	0	2	9	0
T4	0	1	0	5

**Table 4:** N staging according to the reference and PET/MR

PET/MR	Reference			
	N0	N1	N2	N3
N0	23	4	1	0
N1	4	3	1	0
N2	1	0	6	0
N3	0	2	0	5

**Table 5:** M staging according to the reference and PET/MR

PET/MR	Reference		
	M0	M1a	M1b
M0	40	1	1
M1a	0	1	1
M1b	0	0	6

**Fig. 3:**
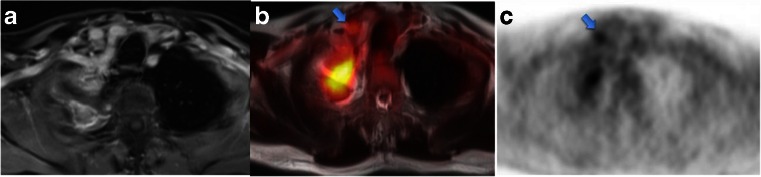
Axial CE MR image (**a**), PET/MR fusion image (**b**) and PET image (**c**) in a 38-year-old man (*arrows* moderately FDG-avid supraclavicular lymph node)

**Fig. 4:**
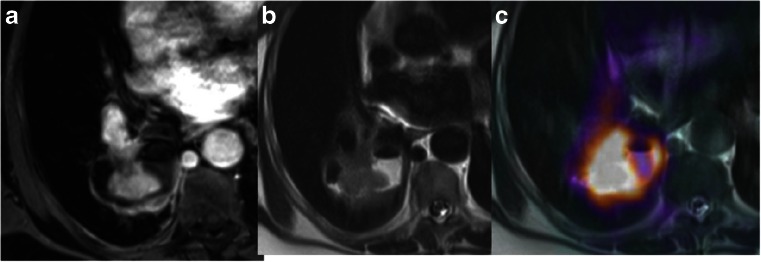
Axial PET/MR images in a 64-year-old woman with lung cancer in the right lower lobe. The MR images (**a**, **b**) and the superimposed PET and MR image (**c**) show a fat plane between the mass and the thoracic vertebra

The T stage of primary tumours was correctly assessed in 37 patients (74 %). Of the remaining 13 patients (26 %), 4 (8 %) were over-staged and 9 (18 %) were under-staged. A total of 1,100 nodal groups were evaluated on PET/MR. Of these nodes, 51 (0.05 %) were classified as malignant, and 63 nodes (0.06 %) were classified as malignant according to the reference standard. Metastatic lesions were confirmed in 18 organs in ten patients (ten bone, three liver, one kidney, three adrenal, one soft tissue). A complete agreement in all findings (T, N and M) with identical nodal and metastasis locations between PET/MR and the reference standard was achieved in 26 patients. Differences in T, N and M staging, but with resectability agreement, were found in 22 patients. Differences in T, N and M staging affecting resectability were found in two patients. In one patient a moderately FDG-avid supraclavicular lymph node was visible on PET/MR images (Fig. 3). No nodal metastases were found in this patient on histological examination. One patient (Fig. 4) presented with a paravertebral lesion, which was considered resectable on PET/MR. In this patient, surgical examination revealed infiltration of the vertebral body.

**Fig. 5:**
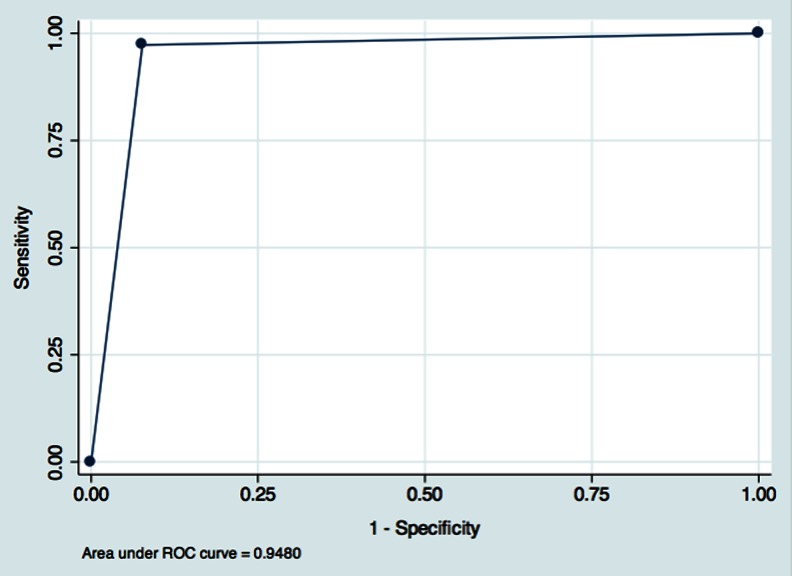
ROC curve and AUC for PET/MR in the assessment of resectability in relation to the reference standard

**Fig. 6:**
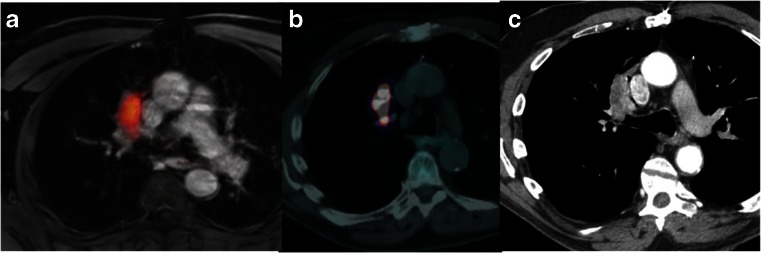
Axial PET/MR image (**a**), PET/CT image (**b**) and CE CT image (**c**) in a 67-year-old man. An irregular nodule adjacent to the superior vena cava shows FDG uptake on both the PET/MR and PET/CT images although separated from the mediastinum by a fat plane

Overall the ROC curve analysis (Fig. 5) showed that PET/MR had a specificity of 92.3 % and a sensitivity of 97.3 % in the determination of resectability with an AUC of 0.95.Intermodality agreement was from substantial to almost perfect for T stage, N stage and M stage (κ 0.627 – 0.823). None of the discrepancies affected clinical management (Fig. 6). PET/MR revealed five suspicious (>5 mm) FDG-avid nodules of the 13 identified on PET/CT. One small minimally avid subsolid lesion not identified on PET/MR was detected on PET/CT and was considered as disease. PET/MR revealed liver metastasis in four patients, that was identified on PET/CT in three. PET/MR detected two non-avid bone lesions that were not seen on PET/CT.

## Discussion

CT and PET are essential imaging techniques for clinical staging before treatment although with the disadvantage of exposure to ionizing radiation. Therefore two separate scans are often performed at different times, potentially resulting in delays in patient evaluation and staging by the local multidisciplinary team. The feasibility of PET/MR as an alternative to PET/CT has recently been demonstrated in a range of diseases, although in limited or heterogeneous cohorts [13–16]. In a comparative evaluation of PET/MR alone versus PET/CT alone in a selected cohort of oncology patients with different cancers, Catalano et al. [18] found that the performance of PET/MR alone more often resulted in a change in clinical management. These authors also acknowledged that in selected anatomical regions, such as the pelvis or head and neck region, the intrinsic contrast resolution of MR allows better delineation of tissue anatomy, including margins, local infiltration, and the relationship of tumours to adjacent structures, than is possible with CT.

PET/MR was recently compared with PET/CT for lung cancer staging in a small pilot study [19]. The new modality was shown to be a potential alternative to PET/CT, with adequate diagnostic image quality. While modality-specific differences were not described, a reference standard was lacking, as this was a feasibility report. Heuch et al. have recently compared a dedicated pulmonary PET/MR protocol and ^18^F-FDG PET/CT in a cohort of 22 patients who received surgery [20]. The authors found that the two modalities had the same accuracy (100 %) in T and N staging compared with histopathology.

In our study we used a combination of surgical information and an enhanced reference standard derived from CT, PET/CT and histopathology to assess the performance of PET/MR in the evaluation of lung cancer resectability. Our results demonstrate a high accuracy of PET/MR with high specificity and sensitivity for this purpose. We found differences between PET/MR and the reference standard. However, clinical management would have been altered in only two patients. In one, disease was considered unresectable owing to suspected N3 disease on PET/MR, whereas the reference standard indicated resectability. In this patient PET/CT was normal. We postulated that an increased FDG uptake might have occurred in benign lymph node with chronic inflammation as reported previously [20]. In the second patient, with a lesion adjacent to the vertebral body, PET/MR images suggest a preserved fat plane, while the disease was unresectable at the time of surgery.

Comparing PET/MR with the reference standard for T staging, we found that the technique slightly overestimated T1 and T2 stages but appeared to underestimate higher stages, i.e. T3 and T4. Our findings are somewhat different from the experience of Yi et al. [12] who reported high diagnostic accuracy of a dedicated whole-body MR protocol for lung cancer staging. However, we decided to use a nondedicated lung protocol (with a shorter acquisition time) and this may have impaired spatial resolution, with a particular impact on delineation of the relationship between tumour and adjacent structures. We found good agreement between PET/MR and the reference standard in N staging with few discrepancies, only one of which was considered to have affected resectability where PET/MR was read as N3 but the node was not malignant on histology. Previous investigators have reported an overestimation of FDG uptake when using PET/MR. Al-Nabhani et al. [14] reported that the performance of PET/MR after PET/CT resulted in delayed tracer accumulation with increasing lesion to background uptake within the lesion, and suggested that this may alter reader perception in assessing nodal disease.

PET/CT has been shown to be associated with a false-positive rate of up to 40 % making histological confirmation of any FDG-avid deposit very important [21]. In our study six patients were understaged by PET/MR. They showed no uptake and were positive on histological examination. In this regard, since PET/MR is a new modality, its interpretation is not as straightforward as for CT or PET/CT and requires a combination of anatomical, metabolic and functional information. In comparison with PET/CT, the MR component might provide better anatomical localization owing to higher resolution with better contrast on T1-weighted imaging, T2 HASTE and DWI/ADC images.

With respect to M staging, PET/MR and the reference standard showed similar results for M0 staging; however, four patients showed M1 staging discrepancies between PET/MR and the reference standard. In one of these patients a skeletal lesion was not avid on PET/MR and was seen only on the follow-up scan. In this patient PET/CT was also normal. One pulmonary nodule judged positive according to the reference standard was probably too small to be characterized by the limited spatial resolution of the PET/MR protocol. This was a 7-mm lesion with a subsolid component. One liver lesion was misinterpreted on PET/MR staging but was recognized on follow-up CE CT.

One of the problems of MR is the lower sensitivity for pulmonary nodule detection compared with the reference methodology, e.g. PET/CT and diagnostic CT. Several studies have evaluated the use of MR for nodule detection and demonstrated the limited value of this technique, which is attributable to the low proton density of lung tissue and the limited spatial resolution [21, 22]. However, current fast T1-weighted gradient-echo sequences have improved the depiction of pulmonary nodules in the range 3 – 5 mm [23, 24]. Even in the presence of cancer, many pulmonary nodules may be benign, and indeed this remains a diagnostic dilemma in oncology.

In this study we tried to reproduce and apply a practical standard MR protocol which consisted of large field of view T1-weighted and T2-weighted sequences, including DWI, plus a whole-body gadolinium CE T1-weighted MR scan combined after PET acquisition in an attempt to reproduce a ‘diagnostic CT protocol’. It has been suggested that the use of contrast media in MR imaging highlights vessels and hilar structures, thereby reducing the diagnostic gap with diagnostic CT for the assessment of mediastinal and chest wall infiltration [25]. However, the optimal diagnostic protocol is still a matter of debate, and particularly the development of new specific dedicated lung protocols may enhance the diagnostic value of the MR component.

In addition to the problem discussed above regarding our reference standard, i.e. not all histology data were available, our study had some other limitations. We did not systematically record quantitative FDG uptake, which would be necessary particularly if we wished to assess the value of the modality for postoperative staging. In addition, we performed PET/MR immediately after PET/CT, and a delay in imaging after injection would have caused image degradation despite partial compensation through an increase in acquisition time; furthermore, accumulation of tracer at more than 90 min after injection may show different distribution characteristics. We recognized that this timing could have caused some bias in the interpretation. However, this approach was done for ethical considerations since PET/CT was always the required clinical examination. It is important to underline that our study aimed to investigate the potential role of PET/MR in assessing lung cancer resectability and we demonstrated that PET/MR results are comparable to published results with PET/CT [26].

Finally, the potential benefits of the individual components of the PET/MR were not assessed and such assessment was beyond the purpose of this study that looked at the value of a “standard” PET/MR protocol, performed similarly to PET/CT to assess tumour resectability. However, the encouraging findings of the current study may form the necessary foundation for further studies, aiming to prove the added value of PET/MR over conventional PET/CT, when including various diagnostic MRI sequences for specific indications. The results of our study indicate the relevance of further investigations of simultaneous PET/MR imaging particularly with respect to the multiparametric role of multiple MR sequences.

### Conclusion

Our results indicate that simultaneous PET/MR imaging may be an alternative to PET/CT performed similarly in staging lung cancer and potentially results in lower radiation exposure. This will need to be balanced by cost and availability constraints.
